# Intensity Trajectories During High-Intensity Interval Training and Their Impact on Health Outcomes in Adolescents: Evidence from School-Based PE Settings (Peer-Heart Studies)

**DOI:** 10.3390/life16060952

**Published:** 2026-06-04

**Authors:** Jarosław Domaradzki, Dawid Koźlenia

**Affiliations:** Department of Biological Principles of Physical Activity, Wroclaw University of Health and Sport Sciences, 51-612 Wroclaw, Poland; jaroslaw.domaradzki@awf.wroc.pl

**Keywords:** high-intensity interval training, school-based settings, training modality, trajectories, exercise intensity

## Abstract

Background: High-intensity interval training (HIIT) is increasingly implemented in school-based physical education, yet little is known about how exercise intensity changes across repeated sessions and whether such trajectories are associated with physiological adaptations in adolescents. Methods: This study, conducted within the PEER-HEART project (ClinicalTrials.gov: NCT06431230), included 145 adolescents from the experimental arms of a broader school-based trial, stratified by sex and training modality: male HIPT (*n* = 24), male HIIT (*n* = 45), female HIPT (*n* = 46), and female HIIT (*n* = 30). The 8-week intervention was delivered twice weekly, and exercise intensity was monitored during 16 sessions using heart rate sensors. Intensity trajectories were examined using visual trajectory plots and slope-based statistical analyses, and pre-to-post changes in body fat percentage, systolic and diastolic blood pressure, and predicted VO_2_max were analyzed. Results: Visual analyses indicated sex- and modality-specific intensity patterns, with the female HIIT group showing the most dynamic trajectory. Mean changes ranged from −0.63 ± 2.37% to −1.54 ± 2.66% for body fat, from −2.73 ± 5.28 to −5.37 ± 4.32 mmHg for systolic blood pressure, from −0.22 ± 5.00 to −2.62 ± 6.25 mmHg for diastolic blood pressure, and from 0.41 ± 3.25 to 3.81 ± 4.67 mL/kg/min for predicted VO_2_max across subgroups. Although most delta values showed no significant main effects, a sex × modality interaction was observed for body fat percentage, and a main effect of sex was observed for predicted VO_2_max. Greater intensity progression was associated with larger reductions in diastolic blood pressure in the female HIIT (β = −708.0, *p* < 0.001, R^2^ = 0.39) and male HIIT groups (β = −377.0, *p* = 0.014, R^2^ = 0.13) and with greater improvements in predicted VO_2_max in the female HIPT (β = 154.0, *p* = 0.029, R^2^ = 0.104) and male HIIT groups (β = 315.0, *p* = 0.029, R^2^ = 0.106). Conclusions: Individual intensity trajectories may help explain variability in physiological responses to school-based interval training and may provide additional insight beyond group-level comparisons alone.

## 1. Introduction

High-intensity interval training (HIIT) has gained considerable attention as a time-efficient and effective strategy for improving health- and fitness-related outcomes in adolescents, particularly when implemented in school-based physical education settings. Previous studies conducted in children and adolescents have shown that school-based HIIT can improve cardiorespiratory fitness, body composition, and physical activity levels [1,2,3]. HIIT is commonly defined as repeated short bouts of vigorous or near-maximal exercise interspersed with brief periods of rest or low-intensity recovery, designed to elicit high cardiovascular and metabolic stress within a relatively short exercise duration. Meta-analytic evidence has further indicated large effects on cardiorespiratory fitness and moderate effects on body composition in youth populations [1]. In addition, HIIT conducted in school settings has been reported to be more effective than traditional exercise approaches for improving selected indices of neuromuscular performance, anaerobic capacity, and fasting glucose [4]. Because such interventions are typically brief and feasible to implement during regular lessons, HIIT appears particularly well suited for integration into physical education classes [2]. Nevertheless, despite the growing evidence base, further research is needed to better understand both the mechanisms and variability of adaptation to school-based HIIT [5]. Previous studies conducted in children and adolescents have shown that school-based HIIT can improve cardiorespiratory fitness, body composition, and physical activity levels [1,2,3].

School-based HIIT has also shown promising effects on several markers of cardiometabolic health. Beneficial changes have been reported for triglycerides, waist circumference, and moderate-to-vigorous physical activity [6], whereas interventions embedded within the school day have demonstrated moderate effects on vigorous physical activity compared with control conditions [7,8]. Moreover, meta-analyses suggest that HIIT may improve waist circumference, body fat percentage, cardiorespiratory fitness, and insulin resistance in adolescents [5]. Typical school-based HIIT interventions are delivered during physical education classes, often last approximately eight weeks, and are usually performed at intensities exceeding 90% of maximal heart rate [9]. Consistent improvements in cardiorespiratory fitness, assessed by shuttle run performance or predicted VO_2_max, have also been observed in adolescent participants [10,11]. In addition, reductions in body fat percentage and visceral adipose tissue, as well as favorable changes in selected strength-related outcomes and cardiometabolic markers, have been reported following HIIT interventions [10,11]. Overall, the effectiveness of HIIT in improving physical fitness and physical activity levels in adolescents has been consistently supported [2]. However, previous findings suggest that the magnitude of these adaptations may differ according to sex and BMI status, with boys and adolescents with lower BMI potentially showing more favorable responses [12,13]. School-based HIIT programs have also been shown to enhance physical well-being and fitness in adolescents [14] and to improve cardiorespiratory fitness in healthy, overweight, and obese youth populations [15].

Despite these promising findings, most previous studies have focused primarily on average pre-to-post intervention effects. Much less attention has been paid to how exercise intensity changes over the course of repeated training sessions and whether such changes may help explain interindividual differences in adaptation [4,6,7]. In real-world school settings, engagement with interval-based exercise is unlikely to be uniform. Instead, training intensity may fluctuate considerably across sessions and weeks, reflecting individual differences in tolerance, motivation, pacing strategies, and adaptation to the imposed workload. Such variability may be highly relevant, because physiological responses to HIIT are likely influenced not only by the assigned training modality, but also by the manner in which exercise intensity evolves over time [13]. However, to the best of the authors’ knowledge, no previous studies have systematically examined intensity progression over time, referred to here as intensity trajectories, during school-based HIIT interventions, nor have they directly linked these trajectories to changes in health-related outcomes. Previous evidence also indicates that fitness- and skill-based HIIT interventions delivered in physical education can increase cardiorespiratory fitness and moderate-to-vigorous physical activity [16].

Therefore, the present study was designed to address this gap by examining how exercise intensity changed across 16 sessions of school-based HIIT or HIPT (high-intensity power training) in adolescents. Specifically, the aims of the study were: (1) to characterize individual and group-level intensity trajectories across the intervention period; (2) to determine whether these trajectories differed according to sex and training modality; and (3) to assess whether the rate of change in exercise intensity, expressed as the individual intensity slope, was associated with changes in body fat percentage, blood pressure, and VO_2_max. By focusing on training dynamics rather than solely on mean intervention effects, the present study offers a novel perspective on adolescent responsiveness to school-based interval training and may provide additional insight into the physiological relevance of individualized intensity progression.

## 2. Materials and Methods

### 2.1. Study Design and Trial Registration

The present study is a secondary analysis of data derived from the PEER-HEART project, a school-based randomized controlled trial examining the effects of two forms of high-intensity interval training in adolescents. Because the original trial methodology has been described previously [17], only procedures directly relevant to the present trajectory-based analyses are summarized here. The study was registered at ClinicalTrials.gov under the identifier NCT06431230, within the PEER-HEART project (Physical Education dosE Response and Health markErs in Adolescents inteRval Training). The project received financial support from the Polish Ministry of Science and Higher Education under the “Science for Society II” program (project no. NdS-II/SP/0521/2023/01).

### 2.2. Ethical Approval

The study was approved by the Ethics Committee of the Wroclaw University of Health and Sport Sciences (decision no. 33/2018, issued on 31 October 2018). All procedures were conducted in accordance with the ethical principles of the Declaration of Helsinki. Written informed consent was obtained from all participants and their parents or legal guardians prior to participation. Consent was also obtained from school authorities.

### 2.3. Participants

A priori sample size estimation was performed using G*Power 3.1 [18] for the broader PEER-HEART trial. Assuming an effect size of 0.20, α = 0.05, and power = 0.95, the required minimum sample size was 310 participants. The broader PEER-HEART trial initially involved 429 students recruited from two secondary schools, with allocation performed at the class level using a simple randomization procedure. After exclusions related to medical contraindications, refusal to participate, involvement in additional structured sports activities, and attendance below the predefined threshold, 307 adolescents were included in the final trial dataset. No adverse events were reported.

For the present trajectory-based analysis, only participants from the experimental arms were included, because only these participants had session-by-session heart rate data during the intervention. The analytical sample comprised 145 adolescents: 24 males in the HIPT group, 45 males in the HIIT group, 46 females in the HIPT group, and 30 females in the HIIT group.

### 2.4. Measurements and Outcomes

Detailed measurement procedures have been reported previously [17]. In the present study, the main outcomes of interest were pre-to-post changes in body fat percentage (BF%), systolic blood pressure (SBP), diastolic blood pressure (DBP), and cardiorespiratory fitness expressed as predicted VO_2_max. These outcomes were selected because they constituted the primary health-related endpoints of the PEER-HEART intervention and allowed examination of whether individual intensity trajectories were associated with the magnitude of physiological adaptation.

#### 2.4.1. Anthropometry and Body Composition

Body height was measured to the nearest 0.1 cm using a GPM anthropometer (GPM Anthropological Instruments, DKSH Ltd., Zürich, Switzerland), according to standard anthropometric procedures. Body mass and body fat percentage were assessed using a Tanita Inner Scan V BC-601 analyzer (Tanita Co., Tokyo, Japan). Body mass index (BMI) was calculated as body mass divided by height squared (kg/m^2^). Prior to assessment, participants were instructed regarding the measurement procedure and standard preparation requirements [19,20].

#### 2.4.2. Blood Pressure

Blood pressure was measured using an Omron BP710 Automatic Blood Pressure Monitor (Omron Healthcare, Inc., Hoffman Estates, IL, USA). Participants remained seated at rest for 10 min before the measurements. During this period and throughout the intervals between repeated measurements, participants remained seated quietly, with their back supported and feet placed on the floor. They were instructed not to talk, use mobile phones, eat, drink, or perform any physical activity. Three readings were obtained at 10 min intervals, and the mean value was used for further analyses [3]. This approach was used to reduce single-measurement variability and to obtain a more stable resting blood pressure estimate, consistent with the use of controlled seated rest and repeated blood pressure assessment in exercise-related studies [21,22]. In the present study, SBP and DBP values recorded before and immediately after the intervention were used to calculate delta changes [23].

#### 2.4.3. Cardiorespiratory Fitness

Cardiorespiratory fitness was assessed using the multistage fitness test (MSFT). During the test, participants wore Polar Verity Sense sensors (Polar Electro, Kempele, Finland), which were also used in the intervention to monitor heart rate responses [24]. Predicted VO_2_max was calculated using the equation proposed by Ramsbottom et al. [25]:

VO_2_max = 3.46 × (L + SN/(L × 0.4325 + 7.0048)) + 12.2,

where L represents the final completed level and SN the number of shuttles completed at that level. The present analyses used the change in predicted VO_2_max from pre- to post-intervention.

### 2.5. Intervention Protocol

Before the intervention, participants received standardized instructions and demonstrations of the exercises included in both protocols to ensure that the movements could be performed safely and consistently during physical education classes. The intervention lasted 8 weeks and was implemented twice weekly during regular physical education classes. Two exercise variants were used: high-intensity interval training (HIIT) based on bodyweight exercises and high-intensity power training (HIPT) based on plyometric exercises. Both variants followed a modified Tabata-type structure with progressively increasing training volume. During weeks 1–2, participants completed four 20 s work bouts interspersed with 10 s rest intervals; during weeks 3–4, the number of bouts increased to six; and during weeks 5–8, participants performed eight bouts per session. Each intervention session was preceded by a standardized 10 min warm-up.

The HIPT protocol included plyometric and fast cyclic movements such as ankle hops, burpees, high knees, squat jumps, butt kicks, and mountain climbers, whereas the HIIT protocol comprised non-plyometric bodyweight exercises such as squats, no-jump burpees, lunges, shoulder taps, push-ups, standing trunk rotations, and sit-ups. All sessions were supervised, and participants were encouraged to perform as many repetitions as possible during each work bout. Throughout the intervention, heart rate was monitored using Polar Verity Sense devices, and exercise intensity was expressed relative to maximal heart rate estimated from the MSFT.

### 2.6. Exercise Intensity Trajectories

The present analysis focused on internal exercise intensity achieved across the 16 intervention sessions. For each participant, sessional intensity was expressed as relative heart rate response (%HRmax).

Intensity trajectory was operationally defined as the within-participant pattern of %HRmax values recorded across the 16 intervention sessions. These trajectories were first explored visually using Sankey diagrams, arc diagrams, and spaghetti plots. Sankey diagrams illustrated transitions between broad intensity zones across intervention weeks, whereas arc diagrams summarized transitions between intensity categories across all sessions. Spaghetti plots displayed participant-level trajectories, with subgroup trends smoothed using quadratic functions to describe possible non-linear patterns.

To quantify longitudinal intensity progression at the individual level, a linear slope was calculated for each participant across the 16 sessions. This slope served as an index of the direction and magnitude of change in exercise intensity over time. Positive slope values indicated progressively increasing intensity, whereas negative values reflected declining intensity across sessions. The slope coefficient was therefore treated as a simplified descriptive index of intensity progression, not as a direct physiological marker of adaptation. These individual slope coefficients were subsequently used as predictors in regression models examining associations with changes in BF%, SBP, DBP, and predicted VO_2_max.

### 2.7. Statistical Analysis

All statistical analyses were conducted to evaluate exercise intensity patterns across the intervention and the relationship between individual intensity progression and pre-to-post changes in health-related outcomes. Quantitative variables are presented as means, standard deviations, and 95% confidence intervals (95% CI). Prior to inferential analysis, statistical assumptions were verified. Distribution normality was assessed using the Shapiro–Wilk test, homogeneity of variance using Levene’s test, and sphericity using Mauchly’s test. When the sphericity assumption was violated, Greenhouse–Geisser correction was applied.

To examine differences in pre-to-post changes (Δ values) in BF%, SBP, DBP, and predicted VO_2_max, two-way analysis of variance (ANOVA) was performed with sex and intervention modality as between-group factors. To assess differences in exercise intensity across repeated sessions, one-way repeated-measures ANOVA was used, followed by Bonferroni-adjusted post hoc comparisons when appropriate.

To investigate whether intensity progression was associated with the magnitude of physiological adaptation, separate linear regression models were fitted for each outcome (BF%, SBP, DBP, and predicted VO_2_max) within each subgroup: males HIPT, males HIIT, females HIPT, and females HIIT. In these models, the individual intensity slope served as the predictor and the pre-to-post delta value as the dependent variable. Model estimates included regression coefficients (β), standard errors (SE), *p*-values, and coefficients of determination (R^2^). Statistical significance was set at *p* < 0.05. All analyses were performed using Statistica 13.3 (StatSoft Poland, Kraków, Poland) and R/RStudio v. 4.5.3 (RStudio PBC, Boston, MA, USA). The arc diagrams were prepared in Google Colaboratory using Python 3.10.

## 3. Results

### 3.1. Visual Exploration of Exercise Intensity Trajectories

**Figure 1:**
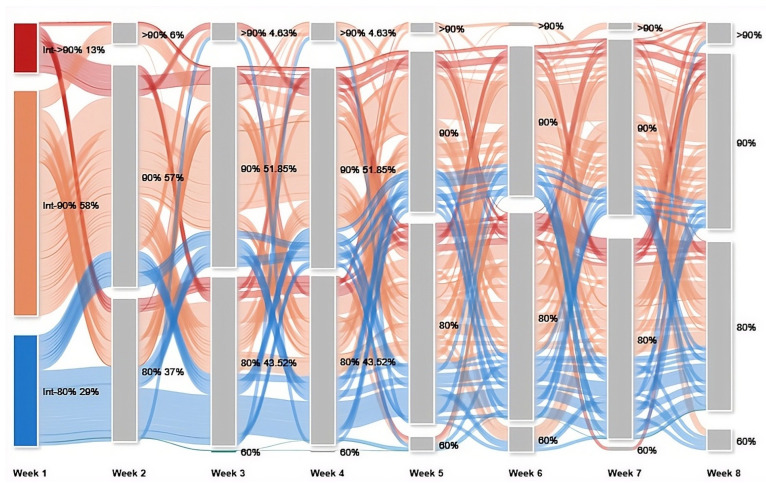
Sankey diagram showing transitions in average exercise intensity across the 8 weeks of the intervention. For clarity, weekly mean intensity values were grouped into four categories: ≤60%, 61–80%, 81–90%, and >90% of HRmax. Each vertical bar represents one intervention week, and the width of each flow corresponds to the number of participants transitioning between adjacent intensity categories. Weekly values were calculated as averages of the two sessions performed within each week.

Visual inspection of exercise intensity across the 8-week intervention indicated a gradual stabilization of training load over time. As shown in Figure 1, most participants achieved and maintained exercise intensities above 80% of HRmax after the second week of the program. The proportion of participants training at >90% HRmax increased during the first 3 weeks and then remained relatively stable, suggesting an early adjustment to the imposed workload. In parallel, the proportion of participants exercising at ≤80% HRmax decreased after Week 2, indicating a shift toward higher relative intensity as the intervention progressed. The most dynamic transitions between intensity categories were observed during the initial phase of the intervention (Weeks 1–3), whereas later weeks were characterized by greater stability. By Weeks 6–8, the majority of participants were concentrated within the 80–90% and >90% HRmax ranges, with only a small number remaining in the lowest intensity category.

**Figure 2:**
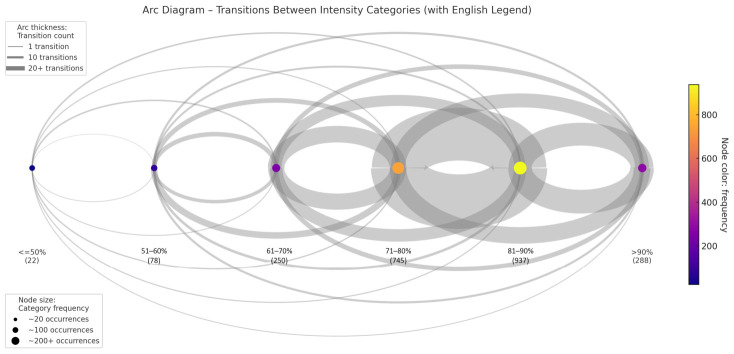
Arc diagram illustrating transitions between exercise intensity categories aggregated across all 16 training sessions. Each node represents a predefined %HRmax category, with node size indicating the frequency of observations within that range. The connecting arcs represent transitions between categories, and line thickness reflects the number of such transitions. The diagram highlights the predominance of moderate-to-high intensity regulation across the intervention period.

A more detailed representation of transitions between intensity zones was obtained using an arc diagram aggregating all session-to-session changes across the intervention period (Figure 2). Each node corresponded to a predefined %HRmax range, and node size reflected the frequency of observations within that category. The most frequently observed zone was 81–90% HRmax (937 occurrences), followed by 71–80% HRmax (745 occurrences) and 61–70% HRmax (250 occurrences). The thickest arcs connected the 71–80% and 81–90% zones, indicating frequent oscillation between these adjacent categories and suggesting that most participants regulated effort within a moderate-to-high intensity range. In contrast, transitions involving the extreme categories (≤60% and >90%) were less frequent, indicating either infrequent exposure to these zones or greater stability once they were reached. Overall, the structure of the diagram suggested convergence toward the 81–90% HRmax zone after the initial adaptation phase, thereby supporting the pattern observed in the weekly Sankey plot.

**Figure 3:**
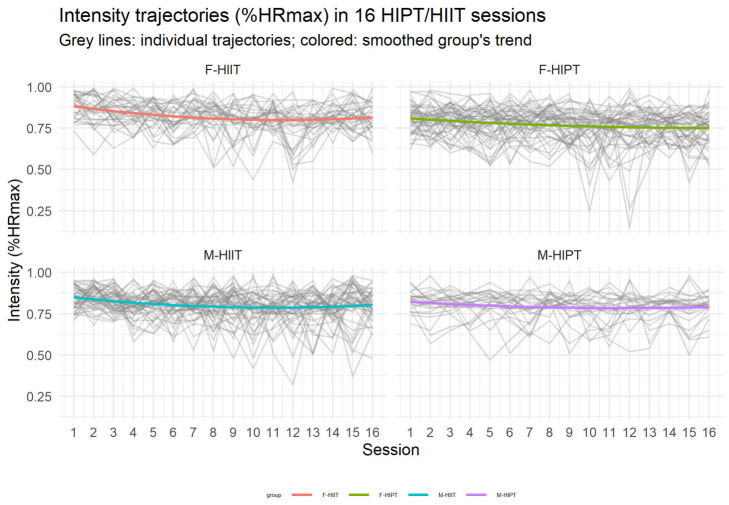
Quadratic-smoothed exercise intensity trajectories (%HRmax) across 16 training sessions in four subgroups defined by sex (female, male) and training modality (HIIT, HIPT). Grey lines represent individual participant trajectories, whereas colored curves represent subgroup-level quadratic trends.

Subgroup-specific patterns of exercise intensity were further examined using participant-level trajectories plotted across 16 sessions with quadratic smoothing applied separately for each sex-by-modality subgroup (Figure 3). The female HIIT group showed the most dynamic pattern, characterized by a U-shaped trend with a decline in relative intensity during the first half of the intervention followed by recovery in the later sessions. This pattern indicates greater temporal variability in the female HIIT group, particularly during the first half of the intervention. In contrast, the female HIPT group displayed a relatively flat trajectory, indicating stable intensity regulation over time. Among males, the HIIT group maintained a consistently high relative intensity throughout the intervention, with a slight upward tendency toward the final sessions, whereas the male HIPT group also demonstrated a stable pattern with minimal fluctuation. Taken together, these subgroup trajectories indicate that intensity progression differed according to both sex and training modality, with the HIIT condition, particularly in females, showing greater temporal variability than HIPT.

### 3.2. Changes in Health-Related Outcomes

Descriptive statistics for pre-to-post changes (Δ) in body fat percentage (BF%), systolic blood pressure (SBP), diastolic blood pressure (DBP), and predicted VO_2_max are presented in Table 1. In the present study, delta values were used primarily for trajectory-based regression analyses examining associations between changes in exercise intensity and physiological adaptation.

Two-way ANOVA showed no statistically significant main effects of sex or training modality for most delta values. However, a significant sex × modality interaction was found for ΔBF% (F = 4.36, *p* = 0.039), indicating that the effect of training modality on fat reduction differed between males and females. Specifically, the HIPT condition was associated with greater fat reduction in females, whereas the HIIT condition appeared more favorable in males. In addition, a significant main effect of sex was observed for ΔVO_2_max (F = 14.15, *p* < 0.001), indicating greater cardiorespiratory fitness gains in males than in females, regardless of training modality. No other differences in delta values reached statistical significance.

### 3.3. Session-by-Session Intensity Analysis

Descriptive statistics for exercise intensity recorded during each of the 16 training sessions are presented in Table 2. One-way repeated-measures ANOVA showed a significant overall effect of session on exercise intensity (F = 8.03, *p* < 0.001), indicating that the relative training load changed across the intervention period. However, Bonferroni-adjusted post hoc comparisons showed no significant differences between the two sessions performed within the same week, suggesting that intensity was relatively stable within weekly microcycles.

To further examine the role of sex and training modality in shaping intensity responses over time, a mixed-effects ANOVA was performed. This analysis revealed two significant interactions: sex × modality (F = 58.23, *p* < 0.001) and modality × time (F = 2.99, *p* = 0.021). These findings indicate that exercise intensity patterns differed between males and females and between training variants and that the effect of training modality was not constant over time. In other words, the evolution of exercise intensity during the intervention differed according to modality. Importantly, no significant differences were observed between paired sessions within the same week in any of the four subgroups (all *p* > 0.05), indicating consistent sessional intensity within weekly training exposure.

### 3.4. Associations Between Intensity Slope and Changes in Body Fat Percentage

To examine whether individual patterns of exercise intensity progression were associated with physiological adaptation, regression analyses were performed using the individual intensity slope across 16 sessions as the predictor and pre-to-post changes in each outcome as the dependent variable.

**Figure 4:**
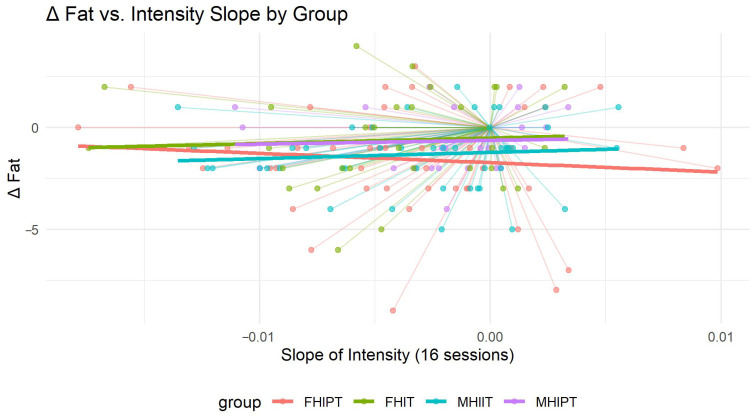
Relationship between individual intensity slope across 16 sessions and pre-to-post change in body fat percentage (ΔBF%) in the four experimental groups. Each point represents one participant, and thick lines represent subgroup-level linear regression models.

The association between intensity slope and ΔBF% is presented in Figure 4 and summarized in Table 3. No statistically significant associations were identified in any subgroup. In the female HIPT group, the regression coefficient was β = −45.8 (SE = 67.1, *p* = 0.498, R^2^ = 0.011). Similarly, no significant relationship was found in the female HIIT group (β = 26.8, SE = 90.4, *p* = 0.769, R^2^ = 0.003), male HIIT group (β = 30.3, SE = 52.3, *p* = 0.564, R^2^ = 0.008), or male HIPT group (β = 18.6, SE = 84.4, *p* = 0.827, R^2^ = 0.002). Across all models, the explained variance was negligible, indicating that intensity progression was not meaningfully associated with changes in body fat percentage.

An interaction model was additionally fitted to determine whether the slope–ΔBF% relationship differed between groups. Using the female HIPT group as the reference category, no significant between-group differences were found for either intercepts or slopes (all *p* > 0.05). Thus, although the subgroup-specific slopes differed in sign and magnitude, these differences were not statistically meaningful.

### 3.5. Associations Between Intensity Slope and Changes in Systolic Blood Pressure

**Figure 5:**
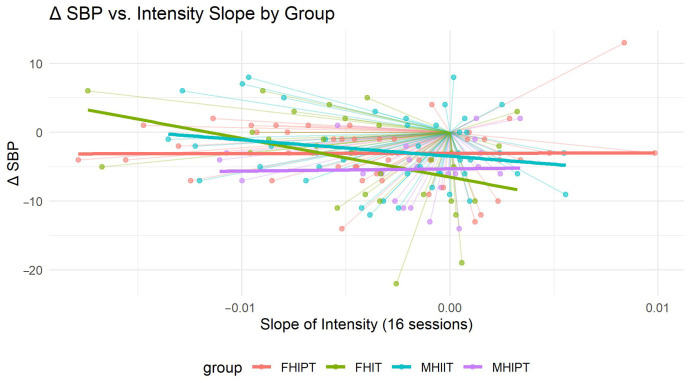
Relationship between individual intensity slope across 16 sessions and pre-to-post change in systolic blood pressure (ΔSBP) in the four experimental groups. Each point represents one participant, and thick lines represent subgroup-level linear regression models.

The relationship between intensity slope and ΔSBP is shown in Figure 5 and Table 3. A statistically significant negative association was observed only in the female HIIT group (β = −561.0, SE = 241.0, *p* = 0.027, R^2^ = 0.162), indicating that participants who showed greater increases in exercise intensity over time tended to demonstrate larger reductions in systolic blood pressure. No significant association was observed in the female HIPT group (β = 4.5, SE = 119.0, *p* = 0.970, R^2^ = 0.00003), male HIIT group (β = −236.0, SE = 163.0, *p* = 0.153, R^2^ = 0.047), or male HIPT group (β = 32.1, SE = 235.0, *p* = 0.893, R^2^ = 0.0008).

The interaction model examining group differences in the slope–ΔSBP relationship was statistically significant overall (R^2^ = 0.098, adjusted R^2^ = 0.052, F(7, 137) = 2.13, *p* = 0.044). Relative to the female HIPT reference group, the female HIIT group showed a significantly steeper negative slope (interaction β = −565.31, *p* = 0.018), indicating a stronger inverse association between intensity progression and SBP change. No significant differences in slope were found for the male HIIT or male HIPT groups. These findings indicate that the female HIIT subgroup showed the clearest relationship between progressive intensity increase and systolic blood pressure reduction.

### 3.6. Associations Between Intensity Slope and Changes in Diastolic Blood Pressure

**Figure 6:**
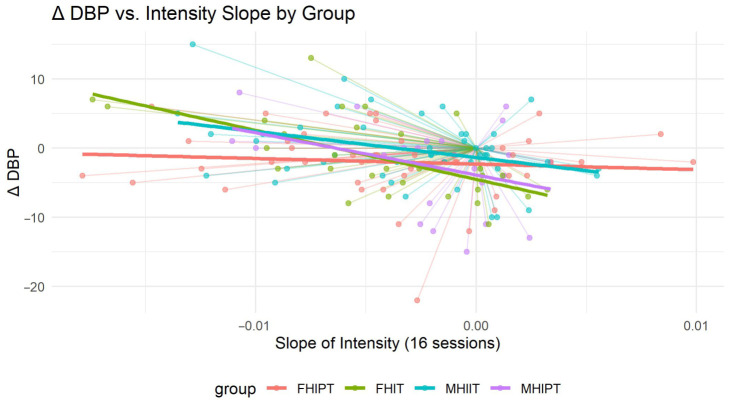
Relationship between individual intensity slope across 16 sessions and pre-to-post change in diastolic blood pressure (ΔDBP) in the four experimental groups. Each point represents one participant, and thick lines represent subgroup-level linear regression models.

The relationship between intensity slope and ΔDBP is presented in Figure 6 and Table 3. Significant negative associations were identified in two subgroups. In the female HIIT group, the association was strong and statistically significant (β = −708.0, SE = 168.0, *p* < 0.001, R^2^ = 0.39), indicating that greater increases in training intensity were associated with larger reductions in diastolic blood pressure. A significant negative association was also observed in the male HIIT group (β = −377.0, SE = 147.0, *p* = 0.014, R^2^ = 0.13). The male HIPT group showed a similar tendency (β = −609.0, SE = 315.0, *p* = 0.066, R^2^ = 0.15), although this effect did not reach the threshold for statistical significance. No significant association was found in the female HIPT group (β = −80.5, SE = 129.0, *p* = 0.535, R^2^ = 0.01).

The interaction model for ΔDBP was statistically significant overall (R^2^ = 0.181, adjusted R^2^ = 0.139, F(7, 137) = 4.31, *p* = 0.0002). Compared with the female HIPT group, the female HIIT group demonstrated a significantly stronger negative slope (interaction β = −627.73, *p* = 0.006), confirming that the relationship between intensity progression and DBP reduction was particularly pronounced in this subgroup. No statistically significant differences in slope were observed for the male HIIT or male HIPT groups, although both showed negative trends.

### 3.7. Associations Between Intensity Slope and Changes in Cardiorespiratory Fitness

**Figure 7:**
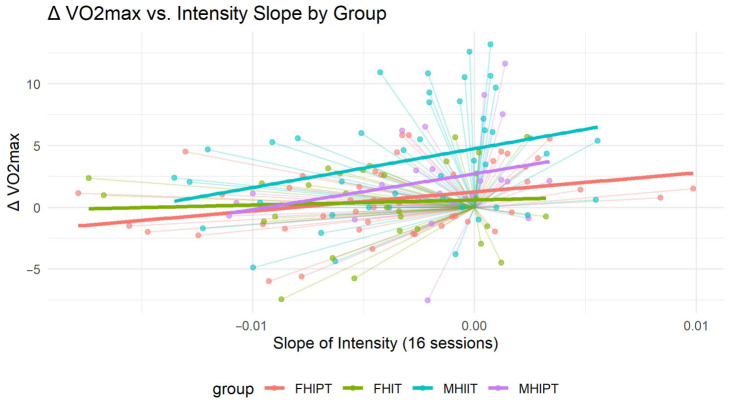
Relationship between individual intensity slope across 16 sessions and pre-to-post change in predicted VO_2_max (ΔVO_2_max) in the four experimental groups. Each point represents one participant, and thick lines represent subgroup-level linear regression models.

The relationship between intensity slope and ΔVO_2_max is shown in Figure 7 and summarized in Table 3. A significant positive association was observed in the female HIPT group (β = 154.0, SE = 68.3, *p* = 0.029, R^2^ = 0.104), indicating that greater increases in training intensity were associated with larger improvements in predicted VO_2_max. A similarly significant association was identified in the male HIIT group (β = 315.0, SE = 139.0, *p* = 0.029, R^2^ = 0.106). No significant relationship was found in the female HIIT group (β = 41.1, SE = 124.0, *p* = 0.743, R^2^ = 0.004), whereas the male HIPT group showed a non-significant positive trend (β = 289.0, SE = 204.0, *p* = 0.170, R^2^ = 0.084).

The group interaction model for ΔVO_2_max was statistically significant overall (R^2^ = 0.206, adjusted R^2^ = 0.166, F(7, 137) = 5.09, *p* < 0.001). However, no significant between-group differences in slope were identified, indicating that the strength of the slope–VO_2_max relationship did not differ significantly across subgroups. The only statistically significant group-level difference concerned the intercept for the male HIIT group (β = 3.50, *p* < 0.001), suggesting greater average VO_2_max improvement in this subgroup even at low or minimal intensity progression.

### 3.8. Summary of Regression Models

Table 3 presents the subgroup-specific regression coefficients, standard errors, *p*-values, R^2^ values, and model fit indices for all outcome variables. Overall, intensity slope was not associated with changes in body fat percentage, but it was significantly related to blood pressure and cardiorespiratory fitness in selected subgroups. The clearest associations were observed for diastolic blood pressure in the female and male HIIT groups and for predicted VO_2_max in the female HIPT and male HIIT groups.

## 4. Discussion

The main finding of the present study is that exercise intensity during school-based interval training did not remain uniform across the intervention period, but followed distinct descriptive patterns that differed according to sex and training modality. Visual analyses showed that most participants converged toward moderate-to-high intensity zones after the initial phase of the program, whereas subgroup-specific plots suggested more dynamic and non-linear patterns in HIIT, particularly in females, and more stable patterns in HIPT. However, these trajectories should be interpreted as descriptive representations of heart rate responses across sessions. The slope-based analyses provided additional exploratory information on the relationship between intensity progression and selected physiological outcomes, but they should not be interpreted as evidence of direct physiological mechanisms [26,27].

The visual analyses provide a useful framework for describing heart rate responses during the intervention. The Sankey diagram indicated that most participants gradually shifted toward intensities exceeding 80% HRmax after the second week, while the arc diagram showed that the most frequent transitions occurred within the 71–90% HRmax range. This pattern suggests that, following a short familiarization period, most adolescents were able to regulate effort within a moderate-to-high intensity domain [6,28,29]. Such stabilization may indicate more consistent exercise engagement across sessions; however, the present study did not directly assess pacing strategy, perceived effort, motivation, or tolerance to repeated interval exercise. These findings are consistent with previous reports showing that school-based HIIT can be implemented with acceptable fidelity and that participants are capable of reaching target intensities during short, teacher-led exercise bouts [5,6,30].

At the same time, the subgroup trajectories indicated descriptive between-group differences. The female HIIT group displayed the most dynamic and non-linear pattern, whereas both HIPT groups showed comparatively stable intensity profiles. The male HIIT group maintained a relatively high intensity throughout the intervention. These findings suggest that exercise intensity patterns varied according to sex and training modality [28,31]. However, the mechanisms underlying these differences remain uncertain, because tolerance to repeated effort, pacing behavior, motivation, perceived exertion, and day-to-day readiness were not directly assessed. Therefore, the more stable pattern observed in HIPT should be interpreted as a descriptive finding rather than evidence of a more consistent external or internal load [30,31].

The quantitative analyses were generally consistent with these visual observations. Although most delta values did not reveal significant main effects of sex or modality, interaction effects indicated that changes in body fat percentage and VO_2_max differed according to sex and training form. Specifically, body fat reduction appeared more favorable in females following HIPT and in males following HIIT, whereas VO_2_max improvements were greater in males regardless of modality. These findings support the view that average intervention effects may obscure subgroup-specific responses [28,31]. They also align with previous work suggesting that the magnitude of adaptation to school-based HIIT may differ according to sex and baseline characteristics [3,13]. Thus, the present findings extend the current literature by showing that sex- and modality-dependent differences may be reflected not only in final outcomes, but also in descriptive patterns of intensity regulation during the intervention itself [26,31,32].

An important contribution of the present study is that it moved beyond average sessional intensity and examined whether individual intensity progression was associated with selected physiological outcomes. This slope-based approach provided a simplified summary of individual intensity progression across the intervention; however, the subgroup regression results should be interpreted as exploratory rather than confirmatory. This is particularly important because some subgroups were relatively small, the number of regression models was high, and several statistically significant associations explained only a limited proportion of outcome variance. No meaningful associations were found between intensity slope and changes in body fat percentage in any subgroup, suggesting that adiposity-related outcomes may be less sensitive to the pattern of intensity progression than cardiovascular or fitness-related variables within the timeframe of the intervention [33]. Body fat reduction in adolescents is likely influenced by multiple factors not captured in the present models, including diet, habitual physical activity outside the intervention, maturation status, and baseline adiposity [26,34]. Therefore, the absence of a clear slope–fat relationship should not be interpreted as evidence that training intensity is unimportant for body composition, but rather that short-term change in body fat is probably determined by a broader set of interacting factors [26].

In contrast, blood pressure outcomes showed clearer, although subgroup-specific, associations with intensity progression. For systolic blood pressure, a significant inverse relationship was observed only in the female HIIT group, whereas for diastolic blood pressure significant inverse associations were found in both female and male HIIT groups. These findings suggest that progressive exposure to higher relative intensity may be related to blood-pressure responses in adolescents participating in HIIT. However, because vascular function was not directly assessed and the regression models were fitted within relatively small subgroups, this interpretation should be treated cautiously. The absence of similarly robust effects in HIPT may indicate that the cardiovascular stimulus generated by increasing intensity over time was more apparent in the HIIT condition than in the plyometric condition [26]. Nevertheless, this modality-specific interpretation remains speculative and requires confirmation in studies designed to compare internal and external load responses directly [33,34].

The results for predicted VO_2_max further suggest that individualized intensity progression may be relevant to cardiorespiratory fitness changes in selected subgroups. A significant positive association between intensity slope and VO_2_max change was found in the female HIPT and male HIIT groups, indicating that participants who increased training intensity more consistently across sessions tended to achieve greater improvements in predicted cardiorespiratory fitness. This observation is compatible with the general principle that a progressive increase in training stimulus may support aerobic adaptation [26,28], but the present slope measure should be interpreted only as a heart rate-derived indicator of intensity progression. Importantly, however, the relationship was not uniform across all groups, which reinforces the idea that the same increase in relative intensity may not produce equivalent physiological benefits in all adolescents [28,31]. Thus, the observed subgroup-specific associations should be interpreted cautiously and require confirmation in studies controlling for additional biological and behavioral factors [27,31,35].

The present findings are broadly consistent with previous literature on the implementation and effects of school-based HIIT. Process evaluations have shown that such programs can be delivered during physical education classes with moderate-to-high fidelity, although maintaining engagement and quality over time remains a challenge [5]. Earlier studies have also shown that not all students consistently achieve the intended heart rate thresholds, even when overall session quality is acceptable [6]. In this context, the present study adds an important dimension by demonstrating that variation in achieved intensity across time is not merely a source of noise, but may contain practically relevant information about individual exercise regulation [26,28]. Rather than relying only on average heart rate across the intervention, examining trajectories and slope-based indices may help describe individual patterns of exercise intensity regulation and indicate which participants may require closer monitoring or individualized adjustment.

From a practical perspective, the results suggest that students can achieve and maintain moderate-to-high exercise intensity within short interval-based formats integrated into regular lessons, but also that intensity should not be treated as a static feature of the intervention [5,6,30]. Monitoring individual intensity trajectories may help teachers and practitioners better understand descriptive patterns of exercise engagement and intensity regulation, particularly because a uniform progression model may not be optimal for all students [25,31]. This may be especially important in real-world school settings, where variability in motivation, readiness, and exercise tolerance is unavoidable [29].

The main limitations of the study include its secondary-analytic character restricted to the experimental groups, the indirect estimation of VO_2_max, the use of heart rate-derived intensity, and the lack of control for factors such as dietary intake, habitual physical activity outside school, sleep, motivation, and biological maturation. Heart rate responses in adolescents may also be influenced by psychosocial stress, hydration status, environmental conditions, accumulated fatigue, and day-to-day readiness, which were not directly assessed in the present study. Another important limitation is the relatively small and unequal subgroup sample sizes, particularly in the male HIPT group, which may have reduced statistical power and affected the stability of subgroup-specific regression estimates. Because several regression models were fitted, isolated significant findings should be interpreted cautiously and treated as exploratory. In addition, the use of linear slope coefficients, although informative, may not fully capture more complex non-linear patterns of adaptation. Accordingly, slope values should be interpreted as simplified descriptive indicators of heart rate-derived intensity progression rather than direct markers of physiological adaptation. The observed associations should therefore be considered hypothesis-generating and require confirmation in larger studies with models adjusted for additional biological, behavioral, and environmental covariates. The study was conducted in school settings; however, the potential influence of school location or broader geographical environment, such as rural versus urban context, was not examined and should be considered in future research. Despite these limitations, the study offers ecologically relevant insight into how individual intensity trajectories may relate to selected physiological responses during school-based interval training in adolescents, but these relationships should be interpreted cautiously due to the exploratory nature of the analyses.

## 5. Conclusions

The present study showed that exercise intensity trajectories during an 8-week school-based interval training program differed according to sex and training modality. Most participants gradually converged toward higher relative intensity zones after the initial phase of the intervention, although HIIT, particularly in females, was characterized by more dynamic and non-linear patterns, whereas HIPT showed greater stability across sessions. While group-level changes in body fat percentage, blood pressure, and predicted VO_2_max were not uniformly significant, individual intensity progression was associated with selected physiological outcomes. In exploratory subgroup analyses, steeper increases in training intensity were associated with greater reductions in selected blood pressure outcomes and greater improvements in predicted VO_2_max in some subgroups, particularly within the HIIT condition. These findings indicate that individual patterns of heart rate-derived intensity progression may provide additional insight into training responsiveness beyond mean group comparisons. However, because the subgroup analyses were based on relatively small samples and several models explained only a limited proportion of variance, the findings should be interpreted cautiously and considered hypothesis-generating. From a practical perspective, the results support the use of individualized intensity monitoring in school-based exercise programs and suggest that sex- and modality-specific intensity patterns should be considered when designing and evaluating interval training interventions in adolescents. Future studies should further examine the contribution of additional factors, including diet, sleep, motivation, and biological maturation, to better explain interindividual variability in training response.

## Figures and Tables

**Table 1:** Descriptive statistics for pre-to-post changes (Δ) in body fat percentage, systolic blood pressure, diastolic blood pressure, and predicted VO_2_max according to sex and training modality.

	Male	Female
HIPT		
Variable	mean ± SD (95% CI)	mean ± SD (95% CI)
ΔFat [%]	−0.67 ± 1.55 (−1.32–−0.01)	−1.54 ± 2.66 (−2.33–−0.75)
ΔSBP [mmHg]	−5.37 ± 4.32 (−7.2–−3.55)	−3.07 ± 4.7 (−4.46–−1.67)
ΔDBP [mmHg]	−2.62 ± 6.25 (−5.27–0.02)	−2 ± 5.09 (−3.51–−0.49)
ΔVO_2_max [mL/kg/min]	2.14 ± 3.91 (0.49–3.79)	0.67 ± 2.84 (−0.17–1.52)
HIIT		
ΔFat [%]	−1.31 ± 1.66 (−1.81–−0.81)	−0.63 ± 2.37 (−1.52–0.25)
ΔSBP [mmHg]	−2.73 ± 5.28 (−4.32–−1.15)	−4 ± 6.89 (−6.57–−1.43)
ΔDBP [mmHg]	−0.22 ± 5 (−1.72–1.28)	−1.37 ± 5.63 (−3.47–0.74)
ΔVO_2_max [mL/kg/min]	3.81 ± 4.67 (2.41–5.21)	0.41 ± 3.25 (−0.8–1.63)

**Table 2:** Descriptive statistics for exercise intensity (%HRmax) recorded during each of the 16 intervention sessions. *p*-values refer to Bonferroni-adjusted post hoc comparisons between the two sessions performed within the same week following one-way repeated-measures ANOVA.

Variable	Mean	−95% CI	95% CI	SD	*p*-Value
W1 s1 (1)	0.84	0.82	0.85	0.08	n.s.
W1 s2 (2)	0.83	0.81	0.84	0.09	
W2 s1 (3)	0.82	0.81	0.83	0.08	n.s.
W2 s2 (4)	0.80	0.79	0.81	0.08	
W3 s1 (5)	0.80	0.79	0.82	0.10	n.s.
W3 s2 (6)	0.79	0.78	0.81	0.09	
W4 s1 (7)	0.80	0.78	0.81	0.09	n.s.
W4 s2 (8)	0.80	0.78	0.82	0.10	
W5 s1 (9)	0.79	0.77	0.81	0.10	n.s.
W5 s2 (10)	0.77	0.75	0.79	0.12	
W6 s1 (11)	0.78	0.76	0.80	0.10	n.s.
W6 s2 (12)	0.77	0.74	0.79	0.13	
W7 s1 (13)	0.78	0.76	0.79	0.10	n.s.
W7 s2 (14)	0.79	0.78	0.81	0.08	
W8 s1 (15)	0.78	0.76	0.80	0.12	n.s.
W8 s2 (16)	0.79	0.77	0.80	0.09	

Abbreviation: n.s.—non-significant.

**Table 3:** Summary of subgroup-specific linear regression models examining the association between individual intensity slope across 16 sessions and pre-to-post changes in body fat percentage (ΔBF%), systolic blood pressure (ΔSBP), diastolic blood pressure (ΔDBP), and predicted VO_2_max (ΔVO_2_max). The table presents regression coefficients (β), standard errors (SE), *p*-values, coefficients of determination (R^2^), and model fit indices (AIC and BIC).

Variable	Group	β	SE β	*p*-Value	R-Squared	AIC	BIC
BFP	FHIPT	−45.80	67.10	0.498	0.01	225.00	230.00
	FHIIT	26.80	90.40	0.769	0.00	142.00	146.00
	MHIIT	30.30	52.30	0.564	0.01	178.00	184.00
	MHIPT	18.60	84.40	0.827	0.00	94.10	97.60
SBP	FHIPT	4.50	119.00	0.970	0.00	278.00	283.00
	FHIIT	−561.00	241.00	0.027	0.16	201.00	205.00
	MHIIT	−236.00	163.00	0.153	0.05	280.00	286.00
	MHIPT	32.10	235.00	0.893	0.00	143.00	147.00
DBP	FHIPT	−80.50	129.00	0.535	0.01	285.00	290.00
	FHIIT	−708.00	168.00	<0.001	0.39	179.00	183.00
	MHIIT	−377.00	147.00	0.014	0.13	271.00	277.00
	MHIPT	−609.00	315.00	0.066	0.15	157.00	161.00
VO_2_max	FHIPT	154.00	68.30	0.029	0.10	227.00	232.00
	FHIIT	41.10	124.00	0.743	0.00	161.00	165.00
	MHIIT	315.00	139.00	0.029	0.11	266.00	272.00
	MHIPT	289.00	204.00	0.170	0.08	136.00	140.00

FHIPT—female high-intensity power training; FHIIT—female high-intensity interval training; MHIIT—male high-intensity interval training; MHIPT—male high-intensity power training; BFP—body fat percentage; SBP—systolic blood pressure; DBP—diastolic blood pressure; VO_2_max—predicted maximal oxygen uptake.

## Data Availability

The data presented in this study is available on request from the author.
